# A rare case of fulminant sepsis secondary to postpartum pyomyoma caused by *Prevotella bivia*: a case report

**DOI:** 10.1097/MS9.0000000000000287

**Published:** 2023-03-24

**Authors:** Tatsuhiko Ogawa

**Affiliations:** Department of Anesthesiology/Intensive Care Medicine, Kochi Health Sciences Center, Kochi City, Kochi, Japan

**Keywords:** pyomyoma, sepsis, fertility, case report

## Abstract

Pyomyoma is rare but can cause life-threatening sepsis from uterine leiomyoma infection. Curative radical surgery to completely remove all infectious foci is preferable if conservative treatment fails, but for patients with fertility concerns, alternatives to uterine removal should be considered. The author reports a case of postpartum pyomyoma to remind clinicians of this rare disease and the need for rapid intervention to preserve patient fertility.

**Case presentation:**

A postpartum female with a fever of unknown origin was admitted to a public hospital. The patient’s general condition rapidly worsened, and surgical removal of the pyomyoma was assumed to be necessary for controlling the infection source. The patient initially refused surgery, as she had fertility concerns; however, she developed septic shock and acute respiratory distress syndrome. Subsequently, surgical intervention was considered imperative, and the patient consented to surgery. Normal uterus was carefully differentiated from degenerated intramural pyomyoma, and the endometrium remained intact. In the pyomyoma specimen, *Prevotella bivia*, an endogenous anaerobic bacterium that can colonize the lower genital tract, was detected.

**Clinical discussion:**

For patients with postpartum sepsis and leiomyoma, pyomyoma should be considered, even if the patient is immunocompetent and has no risk factors. Pyomyoma can be exacerbated into a fulminant and fatal course after subacute, insidious progression.

**Conclusion:**

Comprehensive treatment strategies, including source control of infection and uterine preservation, are required for future fertility. Strict vigilance and appropriate and prompt surgical intervention when conservative treatments fail are crucial to save the patient and preserve fertility.

Highlights

- Postpartum pyomyoma can cause septic shock in previously healthy young females.
- This is the first known pyomyoma due to *Prevotella bivia*, an endogenous bacterium.
- For future pregnancies, infection control and uterine preservation are needed.
- Pyomyoma can worsen to a fatal fulminant course after insidious progression.
- For rapidly progressive cases, close monitoring and prompt intervention are vital.

## Introduction

Pyomyoma is rare but can cause life-threatening sepsis from uterine leiomyoma infection^1^–^3^.

The general treatment for pyomyoma consists of aggressive antibiotics and surgical intervention to control the source of infection^1^–^3^.

However, there are few case reports that determine the definitive treatment for pyomyoma, and particularly for patients of reproductive age who are concerned about fertility, alternative strategies should be considered rather than simply removing the uterus.

We report a case of postpartum pyomyoma to remind clinicians of this rare disease and the need for rapid intervention to preserve patient fertility.

This work has been reported in line with the SCARE criteria^4^.

## Patient perspective

I feel relieved to hear that my uterus was preserved.

### Case presentation

A 29-year-old Asian female who had just experienced her first pregnancy was referred to a public hospital at 8 weeks postpartum with a fever of unknown origin that had persisted for 2 weeks.

A leiomyoma was incidentally confirmed during her pregnancy, although it did not cause any difficulties, and the patient delivered a healthy baby vaginally after preterm rupture of the membranes at 37 gestational weeks. Additionally, the patient had been treated for bacterial vaginosis in the first trimester. Otherwise, the patient did not have any medical problems, drug history, psychosocial history, or pertinent family health history, including any relevant genetic information. Her vital signs were as follows: temperature, 37.1°C; pulse, 107/min; blood pressure, 91/59 mmHg; and respiratory rate, 22/min. Slight abdominal tenderness was intermittently recognized where the known leiomyoma was located during the pregnancy, but there was no rebound tenderness. Physical examinations of the other parts were unremarkable.

Her blood examination showed leukocytosis, and high levels of inflammatory reactions, procalcitonin, and hepatobiliary system enzymes were confirmed. The chest radiography, urinalysis, and vaginal discharge were unremarkable.

**Figure 1:**
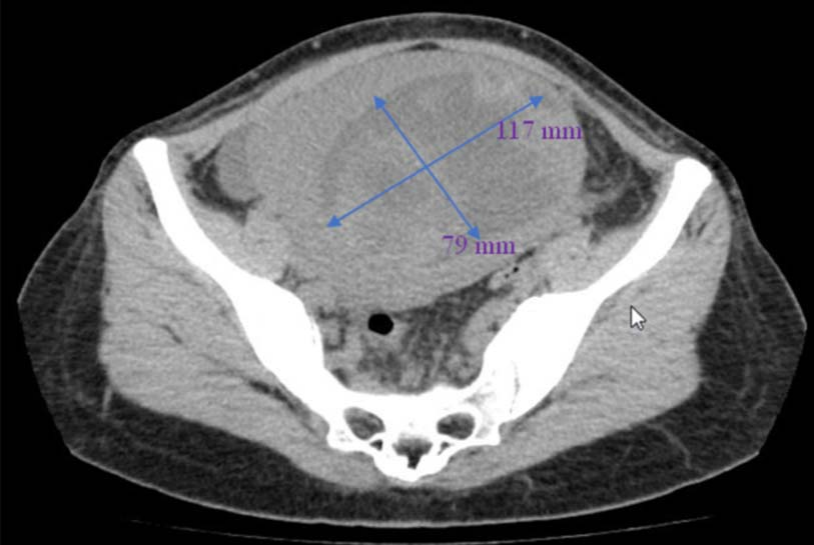
Computed tomography image of a heterogeneous structure (117×79 mm) in the uterus.

A transvaginal ultrasound showed a mixed echogenic mosaic image in the lower abdomen. Computed tomography revealed a heterogeneous structure (117×79 mm) in the uterus (Fig. 1).

Pyomyoma was suspected; however, the patient’s subjective symptoms were not as obvious, and conservative treatment with broad-spectrum intravenous antibiotics (tazobactam/piperacillin) was continued after admission to the general ward.

The next day, the patient’s blood pressure dropped, and she became hypoxic. She was transferred to the ICU for continuous surveillance of her deteriorating general status. Lactated Ringer’s solution helped to increase her blood pressure, and her hypoxia was improved by high-flow nasal cannula oxygen therapy. The patient’s condition was septic, and we proposed that the patient undergo a myomectomy or total hysterectomy to remove the pyomyoma; however, the patient was concerned about the influence of the procedure on her fertility and refused surgery. Considering the patient’s worsening condition, the antibiotics were changed to more broad-spectrum drugs (meropenem and vancomycin).

Six hours later, the patient suddenly presented with shivering and tachypnea. The dramatic progression of severe hypoxia (PaO_2_/FiO_2_ was below 100) and elevation of serum lactate (2.8 mmol/l) were confirmed, and her condition progressed to septic shock. The patient could not maintain oxygenation even if she inspired 100% oxygen with a high-flow nasal cannula, and tracheal intubation was performed. Acute respiratory distress syndrome (ARDS) due to sepsis was diagnosed by bilateral infiltration shadows on a chest radiography and normal left ventricular wall motion without any other abnormal findings that could elevate left atrial pressure.

Discussion by a multidisciplinary staff, including intensive care physicians, gynecologists, and anesthesiologists, led to the conclusion that antibiotics alone could not treat the patient’s pyomyoma and that laparotomy was inevitable to completely remove the infectious source. After receiving consent from the patient, board-certified obstetric experts performed the laparotomy.

**Figure 2:**
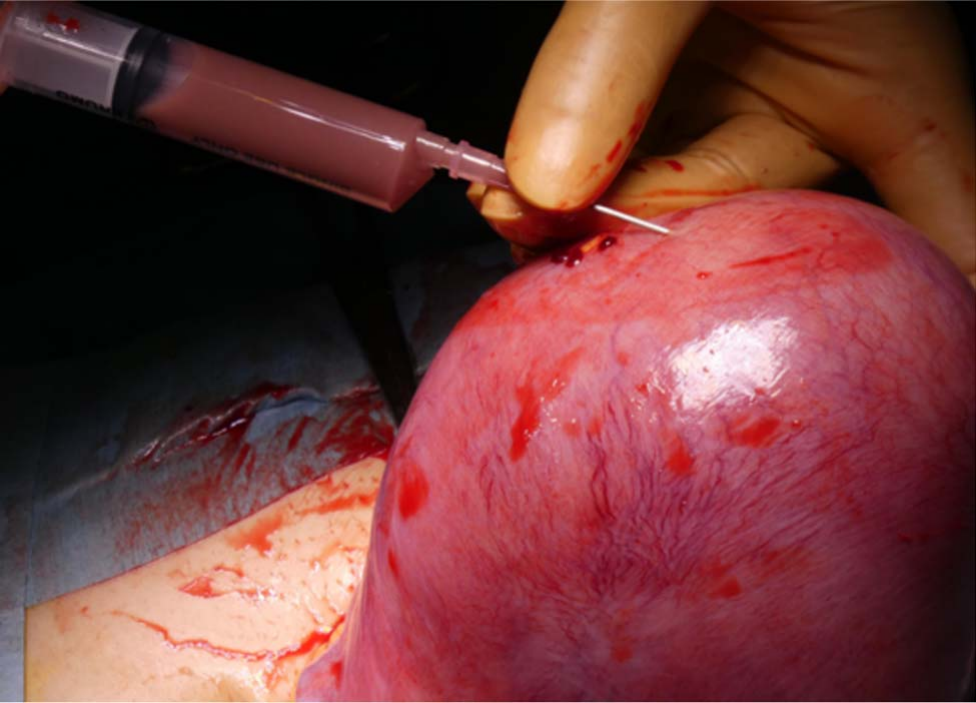
A creamy dark red fluid was drained from the pyomyoma.

**Figure 3:**
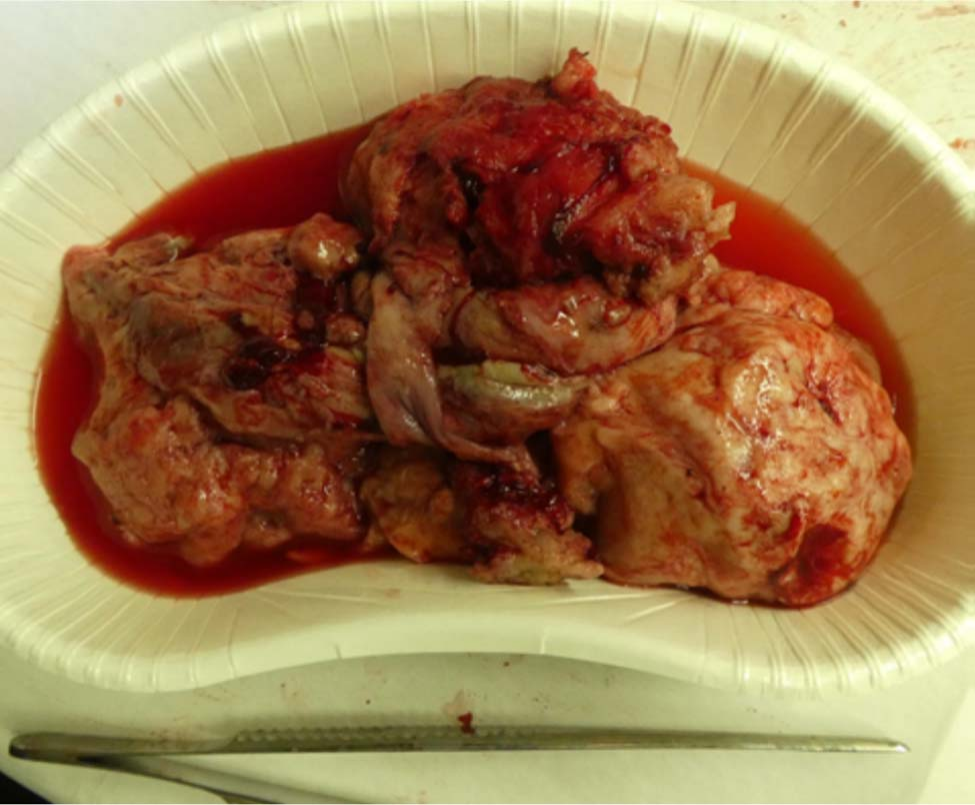
Degenerated pyomyoma after extraction.

Intraoperatively, a degenerating intramural leiomyoma was confirmed. After puncturing the leiomyoma, we suctioned a creamy dark red fluid and submitted it to the laboratory (Fig. 2). Careful manual differentiation of the normal uterus from the degenerating pyomyoma facilitated a myomectomy (Fig. 3), and the endometrium was intact and unscathed. The other region of the uterus was normal; therefore, we concluded that the patient’s fertility could be expected to be preserved.

**Figure 4:**
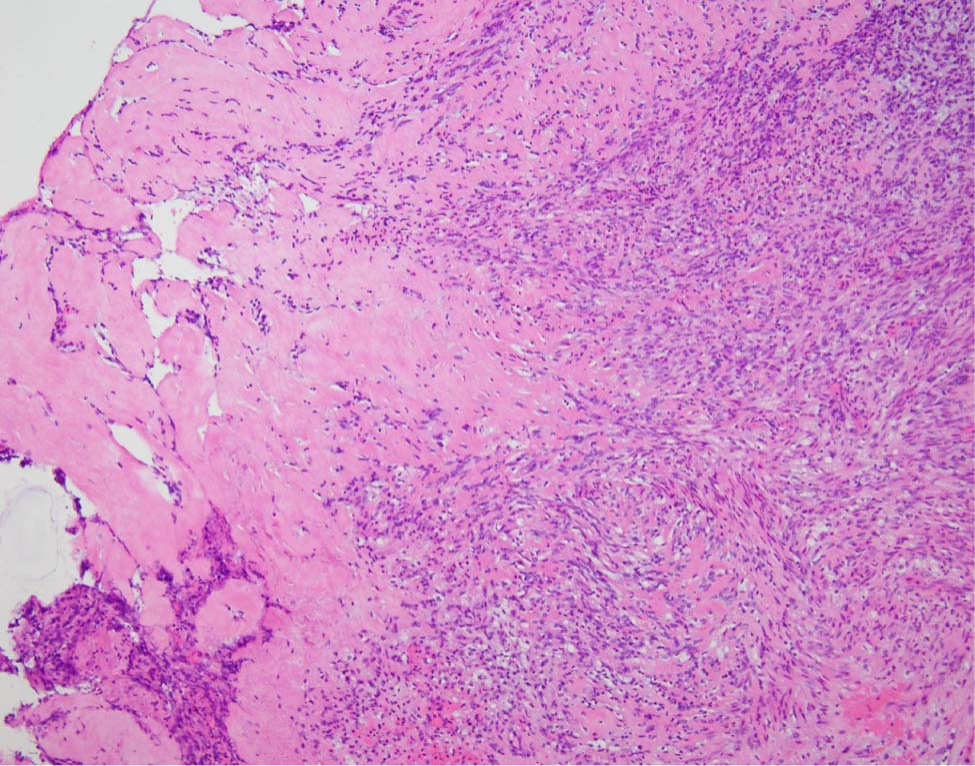
Necrotic hyaline degeneration (histopathological examination).

**Figure 5:**
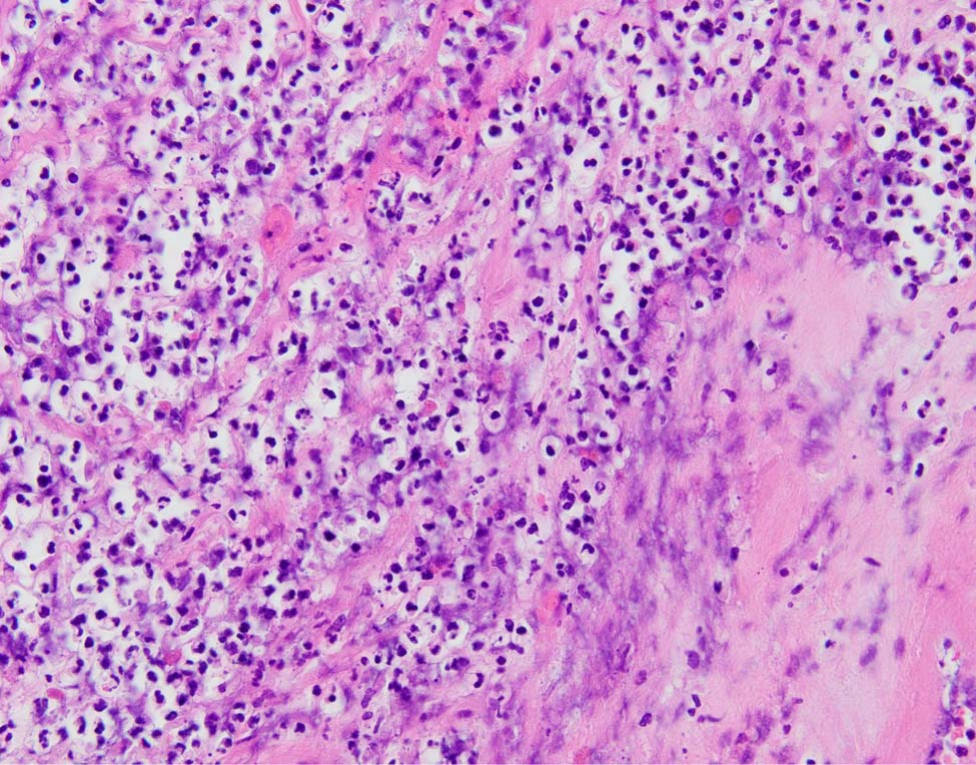
Peripheral intrusion of neutrophils (histopathological examination).

A histopathologic examination revealed a well-defined, circumscribed tubulous tumor and necrotic hyaline degeneration (Fig. 4). Inside the leiomyoma, a proliferative bacterial layer and peripheral intrusion of neutrophils were confirmed (Fig. 5).

All perioperative blood cultures and rapid strep tests of the pharynx were negative.

Only *Prevotella bivia* (*P. bivia*) was cultured from the suppurative fluid obtained during surgery. Antimicrobial susceptibility tests showed that *P*. *bivia* was resistant to ampicillin, benzylpenicillin, cefepime, erythromycin, vancomycin, and levofloxacin but susceptible to sulbactam/ampicillin, ceftazidime, flomoxef, meropenem, ceftriaxone, cefmetazole, imipenem/cilastatin, and clindamycin.

The postoperative course was complicated due to ARDS; however, owing to respiratory physical therapy, the patient’s lung condition gradually improved. In addition, the patient recovered well and was discharged without any other complications.

The patient underwent outpatient examination regularly for postnatal and postoperative follow-up as instructed. The patient is living a generally healthy life, and normal menstruation was confirmed at the 2-month follow-up after discharge.

## Clinical discussion

A leiomyoma that is ischemic or infarcted due to inadequate blood flow in patients with hypertension, diabetes, or atherosclerotic vascular disease is more vulnerable to infection^2^.

Therefore, pyomyoma frequently occurs in the following clinical situations: postpartum or postmenopausal status with vascular disease or following uterine embolization^2^,^5^.

The previous case reports indicate that most infections recorded in the postpartum period developed insidiously over the days to weeks between delivery and the onset of symptoms^6^.

Our patient led a healthy life before conception and did not have any risk factors except for postpartum status. However, bacterial seeding of the leiomyoma may have begun antenatally by direct ascending spread due to bacterial vaginosis and preterm rupture of the membrane. Furthermore, inadequate blood flow to the uterus during the perinatal period may have exacerbated the infection^2^.

The general treatment for pyomyoma consists of aggressive antibiotics and surgical intervention^1^–^3^. Although, curative radical surgery to completely remove all infectious foci is preferable, for patients of reproductive age, it is not a simple matter when considering fertility, and alternative strategies should be considered.

In our case, conservative treatment with broad-spectrum antibiotics failed, and the patient’s condition drastically worsened. Intensive care physicians continued strict vigilance in the ICU and rapidly intervened when the patient developed septic shock and ARDS, at which point surgical intervention was considered to be inevitable. Intraoperatively, normal myometrium was carefully differentiated from a degenerating, infected intramural leiomyoma, and as a result, the endometrium was preserved.

Pinton *et al*.^7^ reported a case of pyomyoma and a successful live birth after a myomectomy. They concluded that a uterine-sparing myomectomy can preserve fertility and remains an important option for women who wish to conceive in the future.

Regarding the causative microorganisms, only *P. bivia*, an endogenous anaerobic bacterium that can colonize the lower genital tract^8^, was detected in the pyomyoma specimen. *P. bivia* is known for being associated with preterm labor, and Strömbeck *et al.* 9 demonstrated that *P. bivia* can invade human cervical epithelial cells *in vitro*. They postulated a possible mechanism by which *P. bivia* escapes host defensive factors, ascends to the uterine cavity, and establishes a subclinical infection^9^.

Additionally, Aroutcheva *et al.* 10 demonstrated that *P. bivia* produces high lipopolysaccharide concentrations and creates a toxic vaginal environment that damages dopamine neurons. They presumed that this condition contributes to the severity of bacterial vaginosis and the development of maternal complications^10^.

Taking their studies into consideration, our case proved that *P. bivia* actually invades the human upper genital tract and causes serious infection *in vivo*. To the best of our knowledge, this is the first report identifying *P. bivia* as the causative organism of pyomyoma and fulminant sepsis.

Various types of infections caused by *P. bivia* have been reported, but there is no consensus on the optimal antimicrobial drugs^11^.

*P. bivia* was shown to be particularly susceptible to metronidazole, imipenem, and piperacillin/tazobactam^12^; however, most *P. bivia* strains are β-lactamase positive^13^.

In our case, tazobactam/piperacillin and meropenem might be effective according to the antimicrobial susceptibility test; however, surgical intervention was eventually necessary due to the progressive deterioration of the general condition.

Prior to the admission date, the patient had been stable, despite exhibiting a high fever and inflammatory reactions. However, the patient’s general condition drastically worsened, leading to septic shock and ARDS the next day.

It is not clear what caused this dramatic change in an originally healthy patient, but we considered that two main factors might have affected the clinical course.

First, the leiomyoma was intramural and did not show local signs. Due to its spatial positioning, the pyomyoma did not cause an abnormal vaginal discharge or lead to perforation of the uterus, which might have caused acute abdomen^14^. Therefore, the infection did not noticeably affect the patient’s condition until it reached an advanced stage.

Second, the patient had a history of bacterial vaginosis. This condition is not rare but can cause maternal complications^10^,^15^. Prior bacterial vaginosis might have led to the insidious growth of *P. bivia* in the patient’s uterus.

This case demonstrates that clinicians should keep in mind that this rare disease can be exacerbated into a fulminant and fatal course after subacute, insidious progression.

Due to close monitoring in the ICU, our multidisciplinary team was able to perform surgery in a timely manner and preserve the uterus. Considering the rapid progression of this disease, strict surveillance and prompt, appropriate surgical intervention were crucial.

## Conclusions

For postpartum sepsis patients with leiomyoma, pyomyoma should be considered, even if the patient is immunocompetent with no risk factors. Pyomyoma can be exacerbated into a fulminant and fatal course after subacute, insidious progression. Strict surveillance and prompt, appropriate surgical intervention are crucial to saving the patient and preserving the uterus.

## Ethical approval

The author affirms that the work described has been carried out in accordance with The Code of Ethics of the World Medical Association (Declaration of Helsinki) for experiments involving humans.

## Consent

Written informed consent was obtained from the patient for the publication of this case report and accompanying images. A copy of the written consent is available for review by the Editor-in-Chief of this journal on request.

## Sources of funding

There are no funding sources for this article.

## Author contribution

T.O. participated in this case, reviewed references, and wrote this manuscript. The author has full responsibility for this article.

## Conflicts of interest disclosure

The authors have no conflicts of interest to declare.

## Research registration unique identifying number (UIN)

None.

## Guarantor

Tatsuhiko Ogawa.

## Provenance and peer review

Not commissioned, externally peer reviewed.
